# Antimyeloma Effects of the Heat Shock Protein 70 Molecular Chaperone Inhibitor MAL3-101

**DOI:** 10.1155/2011/232037

**Published:** 2011-09-29

**Authors:** Marc J. Braunstein, Sadeaqua S. Scott, Craig M. Scott, Shannon Behrman, Peter Walter, Peter Wipf, Jeremy D. Coplan, William Chrico, Danielle Joseph, Jeffrey L. Brodsky, Olcay Batuman

**Affiliations:** ^1^Division of Hematology/Oncology, Department of Medicine, SUNY Downstate Medical Center, Brooklyn, NY 11203, USA; ^2^Department of Biological Sciences, University of Pittsburgh, Pittsburgh, PA 15260, USA; ^3^Department of Biology, Clarion University, Clarion, PA 16214, USA; ^4^Department of Biochemistry and Biophysics, Howard Hughes Medical Institute, University of California, San Francisco, CA 94158, USA; ^5^Department of Chemistry and Center for Chemical Methodologies and Library Development, University of Pittsburgh, Pittsburgh, PA 15260, USA

## Abstract

Multiple myeloma (MM) is the second most common hematologic malignancy and remains incurable, primarily due to the treatment-refractory/resistant nature of the disease. A rational approach to this compelling challenge is to develop new drugs that act synergistically with existing effective agents. This approach will reduce drug concentrations, avoid treatment resistance, and also improve treatment effectiveness by targeting new and nonredundant pathways in MM. Toward this goal, we examined the antimyeloma effects of MAL3-101, a member of a new class of non-ATP-site inhibitors of the heat shock protein (Hsp) 70 molecular chaperone. We discovered that MAL3-101 exhibited antimyeloma effects on MM cell lines *in vitro* and *in vivo* in a xenograft plasmacytoma model, as well as on primary tumor cells and bone marrow endothelial cells from myeloma patients. In combination with a proteasome inhibitor, MAL3-101 significantly potentiated the *in vitro* and *in vivo* antimyeloma effects. These data support a preclinical rationale for small molecule inhibition of Hsp70 function, either alone or in combination with other agents, as an effective therapeutic strategy for MM.

## 1. Introduction

This study explored the cytotoxic effects of MAL3-101, a recently developed inhibitor of Hsp70 [1], on multiple myeloma (MM) tumor growth. MM is a bone marrow (BM) neoplasm of plasma cells and remains incurable [2]. Despite significant improvements in patient outcomes as a result of high-dose chemotherapy with stem cell rescue, and novel therapies with bortezomib, thalidomide, and lenalidomide [3, 4], disease progression in MM leads to mortality resulting from accumulating genetic mutations, prolonged tumor survival, and treatment resistance [5, 6]. Equally important in MM pathogenesis and progression are the tumor enhancing effects of the BM microenvironment [7, 8], particularly the increased neovascularization of the MM niche [9] by endothelial progenitor cells (EPCs) [10]. However, both the tumor and microenvironment in MM are significantly affected by proteasome inhibition via interruption of cell survival pathways [8, 11–13]. The potent antimyeloma effects of bortezomib (PS-341; Velcade), a first-in-class selective inhibitor of the 26S proteasome, are largely due to a cellular stress response characterized by transcription of proteasome subunits and molecular chaperones of the heat shock protein family which include Hsp90 and Hsp70, and their downstream regulators of tumor growth [8, 12, 14–20]. Thus, blockade of molecular chaperones is currently being explored in preclinical studies and clinical trials for their antimyeloma effects, either synergistic with bortezomib or in combination with other agents [4, 21, 22]. 

 MAL3-101 inhibits the ability of Hsp40 cochaperones to stimulate Hsp70 ATPase activity and thereby compromises essential Hsp70 cellular functions [1, 23]. Our rationale for studying the antimyeloma effects of MAL3-101 was fourfold. First, in plasma cells, the Hsp70 homolog in the endoplasmic reticulum (ER), BiP, enhances the folding and secretion of normal and misassembled immunoglobulins (IGs) and prevents their accumulation [24]. Second, Hsp70 expression is upregulated in MM cells [25, 26], and in treatment-resistant MM cell lines [26], and especially after exposure to clinically effective antimyeloma drugs that inhibit other components of the protein quality control machinery [27]. Third, Hsp70 gene expression and overexpression are associated with human cancers [28–32]. Fourth, inhibition of Hsp70 in cancer cells triggers tumor-specific apoptosis and cell death by inhibiting lysosomal membrane permeabilization, a hallmark of stress-induced cell death [33, 34] The latter mechanism was suggested by stabilization of lysosomes via Hsp 70 binding to an endolysosomal anionic phospholipid bis(monoacylglycero)phosphate (BMP), an essential cofactor for lysosomal membrane sphingomyelin metabolism [34]. 

 Hsp70 gene and protein expression are upregulated in MM cells after exposure to bortezomib as well as after application of 17-allylamino-17-demethoxygeldanamycin (17-AAG), which inhibits Hsp90 chaperones [11, 15, 16, 18, 25, 35]. Notably, Hsp70 acts at several nodes in the apoptotic pathway [16, 29, 36], and thus its inhibition may overcome the differential responsiveness to bortezomib as well as the side effects encountered in its use against MM [20, 22, 37]. In turn, inhibition of Hsp72 by small molecule inhibitors was shown to potentiate the *in vitro *apoptotic effects of 17-AAG on MM tumor cell lines [16, 21]. Thus, we reasoned that MAL3-101 might act synergistically and potentiate *in vitro *and *in vivo *the antimyeloma effects of proteasome and Hsp90 inhibitors. 

 In accordance with our premise, we discovered that MAL3-101 exhibits potent inhibitory effects on proliferation and survival of MM cells, including primary tumor cells and EPCs obtained from MM patients. MAL3-101-induced inhibition of MM cell growth was characterized by an arrest of cell cycle progression and activation of the intrinsic apoptotic pathway in these cells. Moreover, strong synergy was found in the *in vitro* cytotoxic effects of MAL3-101 with inhibitors of the proteasome and Hsp90. The synergy between MAL3-101 and proteasome inhibition on MM cell growth was then studied *in vivo *using the xenograft plasmacytoma mouse model [38]. The *in vivo *study reproduced the inhibition of tumor cell growth found *in vitro*. These data suggest that by targeting Hsp70 activity in the tumor and its vascular microenvironment, and by allowing a dose reduction of synergistic agents without compromising effectiveness, Hsp70 inhibition could improve existing strategies by adding to the arsenal of agents that combat MM and prolong survival by circumventing treatment resistance.

## 2. Materials and Methods

### 2.1. Reagents and Cell Cultures

MAL3-101 and MAL3-51 (see Figure S1 in the Supplementary material available online at doi: 10.1155/2011/232037), the proteasome inhibitors MG-132 (A.G. Scientific, San Diego, Calif) and bortezomib (PS-341, LC Labs, Woburn, Mass), and the Hsp90 inhibitor 17-AAG (Calbiochem/EMD Biosciences, San Diego, Calif) were dissolved in dimethylsulfoxide (DMSO; Sigma-Aldrich, St. Louis, Mo) and stored at −20°C. Control cells received vehicle alone (0.03% DMSO). The MM cell lines examined were NCI H929, RPMI-8226, and U266 (ATCC, Manassas, Va). Normal peripheral blood mononuclear cells (PBMCs) and bone marrow (BM) cells were obtained from StemCell Technologies (Vancouver, Canada). Primary MM cells and EPCs were from BM aspirates of newly diagnosed patients following informed consent. MM cells were enriched to >95% CD138^+^ cells by positive selection using anti-CD138 MACS Microbeads according to the manufacturer's instructions (Miltenyi, San Diego, Calif). EPCs were derived from BM aspirates of newly diagnosed patients, maintained in EndoCult medium (StemCell Technologies), and used at first passage as previously described [39, 40]. Cell lines, PBMCs, and BM cells were maintained in RPMI-1640 supplemented with 10% heat-inactivated fetal calf serum as previously described [39, 40].

### 2.2. Cytotoxicity Assays

Cells (1 × 10^5^) were plated in 96-well microtiter plates (Costar, Cambridge, Mass) in 100 *μ*L of growth medium and exposed for the indicated periods to the noted concentrations of compounds. Control cells were cultured in the same volume of 0.03% DMSO. All studies were performed in triplicate and repeated on at least three independent occasions. Survival was measured by an MTS assay (Promega, Madison, Wis) as per the manufacturer's directions. Cell viability was also measured by trypan blue dye exclusion in identically plated cultures. 

 Apoptosis induction in control (DMSO-treated) or drug-treated cells was determined using an Annexin-V-FLUOS Staining Kit according to the manufacturer's instructions (Roche, Indianapolis, Ind). Briefly, cells (1 × 10^5^) were harvested at indicated time points after treatment, and Annexin V-fluorescein isothiocyanate (FITC) and propidium iodide (PI) were added to individual samples and incubated for 30 min in the dark. Fluorescence was analyzed by a FACSort flow cytometer (BD Biosciences, San Jose, Calif) by acquiring 10,000 events per sample.

### 2.3. Cell Cycle Analysis

Cell cycle analysis of control (DMSO-treated) and MAL3-101-treated NCI-H929 cells (2 × 10^5^) was assessed by PI staining and FACS analysis of samples. Resulting DNA distributions were analyzed for the proportions of cells in *G*_0_/*G*_1_ and *G*_2_/*M* phases of the cell cycle after subtractive gating of cell doublets and debris, as previously described [41].

### 2.4. Western Blotting

Whole-cell lysates were prepared using the Mammalian Cell Lysis Kit (Sigma-Aldrich) and analyzed by Western blot analysis. Equal amounts of protein were separated by SDS-PAGE and electrotransferred onto a nylon membrane. Primary antibodies to detect caspase-3 (Cell Signaling Technology, Danvers, Mass), poly-ADP-ribose polymerase (PARP; Abcam, Cambridge, Mass), and *β*-actin (Sigma-Aldrich) were used along with a horseradish peroxidase-conjugated goat-antimouse polyclonal secondary antibody (BD Biosciences, Franklin Lakes, NJ). Enhanced chemiluminescence substrate (Pierce Biotechnology, Rockford, Ill) was used for antibody detection.

### 2.5. Reverse-Transcription Polymerase Chain Reaction (RT-PCR) Analysis of XBP1 mRNA Splicing

Unspliced (XBP-1u, 285 bp) and spliced (XBP-1s, 259 bp) XBP-1 mRNA levels of treated NCI-H929 cells were determined by PCR amplification of total RNA that was reverse transcribed by SuperScript Reverse Transcriptase (Invitrogen, Carlsbad, Calif). PCR primers (5′-TTACGAGAGAAAACTCATGGC-3′ and 5′GGGTCCAACTTGTCCAGAATGC-3′), which flank the 26-nt intron of XBP1 mRNA, were used for PCR amplification with *Ex Taq *polymerase (Takara Bio, Shiga, Japan).

### 2.6. Human Immunoglobulin Light-Chain Enzyme-Linked Immunosorbent Assay (ELISA)

Levels of secreted and intracellular *κ* and *λ* light chains (LCs) were determined using Human Kappa and Lambda (bound and free) ELISA Quantitation Kits (Bethyl Laboratories, Montgomery, Tex) according to the manufacturer's instructions. Pellets and supernatants were obtained from 10^6^ cells cultured in serum-free medium overnight. Total protein in whole-cell lysates from cell pellets and supernatants was determined using the Bio-Rad Protein Assay (Bio-Rad Laboratories, Hercules, Calif), and 500 ng total protein was used in each ELISA. To account for differences in secretion relative to synthesis of LCs between MM cell lines, LC production was assessed as the proportion of secreted versus intracellular LC levels.

### 2.7. Xenograft Plasmacytoma Murine Model

NOD/SCID/IL-2R gamma null (NSG) mice (42–52 days old) were obtained from Jackson Laboratories (Bar Harbor, Me) and maintained under pathogen-free conditions at SUNY Downstate Medical Center animal resources facility. The study was conducted according to protocol approved by the Institutional Animal Care and Use Committee (IACUC) of Downstate Medical Center. Mice were inoculated s.c. into the right flank with 3 × 10^7^ NCI-H929 cells in 100 *μ*L of RPMI 1640, together with 100 *μ*L of Matrigel basement membrane matrix (BD Biosciences, Bedford, Mass) as described by LeBlanc et al. [38]. Mice were then divided into four treatment groups containing 4 mice each receiving vehicle (DMSO and PBS), or 1 mg/kg PS-341 (Bortezomib), or 40 mg/kg MAL3-101, or 1 mg/kg PS-341 and 40 mg/kg MAL3-101. All groups were treated via i.p. injections twice weekly, at the same schedule. Caliper measurements of the longest tumor diameters were performed on the days of treatment to estimate the tumor volume, using the following formula: 4*π*/3 × (width/2)^2^ × (length/2), representing the three-dimensional volume of an ellipse. Animals were sacrificed when their tumors reached 5.5 cm or became necrotic; the earliest sacrifice was one of the vehicle-treated animals on day 20 of treatment. Animal studies were independently replicated, and data from all experiments are shown. 

### 2.8. Statistical Analysis

DMSO-treated cultures were compared to drug-treated cultures by Student's *t-*tests or by Dunnett's *post hoc *test following significant repeated-measures one-way analysis of variance. All tests were two tailed, and statistical significance was set at *P* ≤ .05. Isobologram analyses were performed to derive combination index (CI) values based upon the Chou-Talalay method using CalcuSyn software (Biosoft, Ferguson, Mo and Cambridge, United Kingdom) as described [14]. 

 The *in vivo* component of the study was analyzed with two approaches. First, the extent of plasmacytoma growth was analyzed using a general linear model (GLM) with days of tumor measurements as the repeated measure and treatment condition (vehicle, MAL3-101, PS-341 or PS-341 plus MAL3-101 in combination) as the categorical variable. In the GLM, we adjusted for the two experiments from which the data had been collected (experiments 1 and 2). We examined the statistical analysis for a repeated measure by condition effect to assess the difference between conditions across the days of treatment. ANOVAs for condition effects were performed for each day followed by post hoc Tukey's honest significant difference (HSD) testing. Second, using the GLM analysis, we used the vehicle arm as a reference point to calculate the mean percentage difference for each treatment alone and the combination versus vehicle on tumor growth. Results were adjusted for experiment number. Conditions were compared using post hoc testing with Tukey's HSD testing.

## 3. Results

### 3.1. MAL3-101 Suppresses Growth and Initiates Apoptosis in Human MM Cells

**Figure 1:**
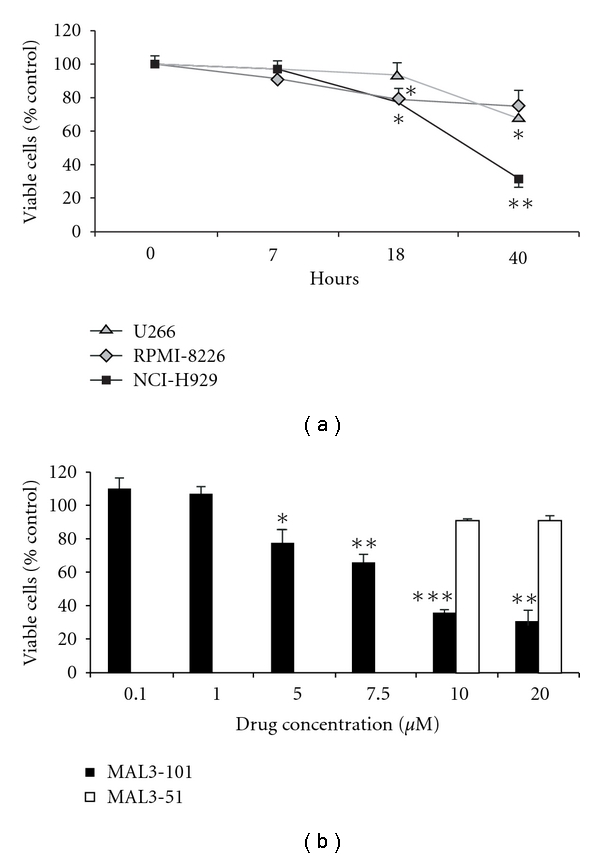
MAL3-101 induces a graded cytotoxic effect in multiple myeloma (MM) cell lines. (a) MM cell lines (1 × 10^5^) were exposed to 10 *μ*M MAL3-101 for the indicated culture periods, and the fold change in percent viable cells in treated versus control cells was determined by an MTS assay. (b) NCI-H929 cells were exposed to the indicated concentrations of MAL3-101 or MAL3-51 for 40 h, and the percent viability, as compared to control, DMSO-treated cells, was assessed by an MTS assay. Data are presented as means and SDs from three independent experiments; *P < 0.05, **P < 0.01, and ***P < 0.001.

To determine the effect of MAL3-101 on MM cell lines, NCI-H929, U266, and RPMI-8226 cells were cultured with increasing concentrations of MAL3-101 and harvested at different time points after treatment. Control cells were treated with vehicle (DMSO). The highest level of cytotoxicity, as assessed by an MTS assay, was observed in NCI-H929 cells (Figure 1(a)); dose-response studies at 40 h exposure showed the IC50 to be 8.3 *μ*M (Figure 1(b)). Exposure to MAL3-101 beyond 48 h did not result in an additional increase in cell death or apoptosis (data not shown). In contrast, there was no response to 10 or 20 *μ*M MAL3-51 (Figure 1(b)), a substantially less potent Hsp70 modulator [42]. These data strongly suggest that the cytotoxic effect of MAL3-101 was directly related to its ability to inhibit Hsp70 activity in NCI-H929 cells. A cytotoxic effect of MAL3-101 was also observed in the RPMI-8226 cell line although to a lesser extent than observed on the NCI-H929 cells (Figure 1(a)). 

**Figure 2:**
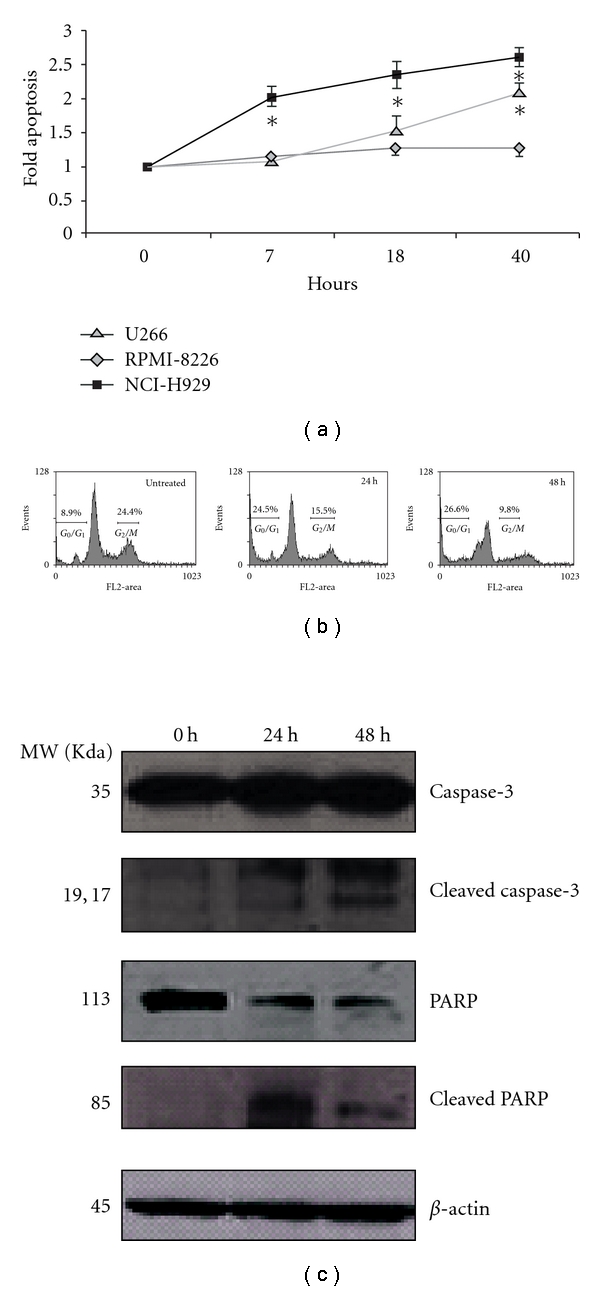
MAL3-101 induces cell cycle arrest and caspase-mediated apoptosis in the multiple myeloma (MM) cell line NCI-H929. (a) MM cell lines were exposed to 10 *μ*M of MAL3-101 for 40 h, and the fold change in apoptosis in MAL3-101-treated versus control, DMSO-treated cells was determined by flow cytometry. Data are presented as means and SDs from three independent experiments; *P < 0.05. (b) The percentages of NCI-H929 cells in the *G*_0_/*G*_1_ and *G*_2_/*M* phases of the cell cycle are indicated in each panel. This result is representative of three independent experiments that yielded similar results. (c) NCI-H929 cells were exposed to 10 *μ*M MAL3-101 for the indicated culture periods and immunoblotted with primary antibodies to detect caspase-3 and poly(ADP-ribose) polymerase (PARP). To ensure equal loading, *β*-actin levels were examined as a control. Bands corresponding to intact and cleaved caspase-3 and PARP are indicated. The data are representative of four experiments.

 Because MAL3-101 initiates apoptosis through its ability to arrest the cell cycle as well as by activating the cleavage of caspase-3 and PARP in breast cancer cells [23], we next examined these features in NCI-H929 cells. FACS analysis showed that exposure to 10 *μ*M MAL3-101 produced a time-dependent increase in apoptosis (Figure 2(a)). This treatment also inhibited cell cycle progression, as indicated by a nearly 3-fold increase in the sub-*G*_0_/*G*_1_ phase and a 2.5-fold decrease in cells in the *G*_2_/*M* phase within 48 h of culture (Figure 2(b)). This result was supported by an immunoblot analysis, which showed a time-dependent increase in the cleavage of caspase-3 and PARP after exposure to MAL3-101 (Figure 2(c)). Taken together, these results indicate that MAL3-101 inhibits MM tumor cell growth and initiates an apoptotic program with substantially greater efficacy in some cell lines than others.

### 3.2. Exposure to MAL3-101 Enhances the Antimyeloma Effects of MG-132

**Figure 3:**
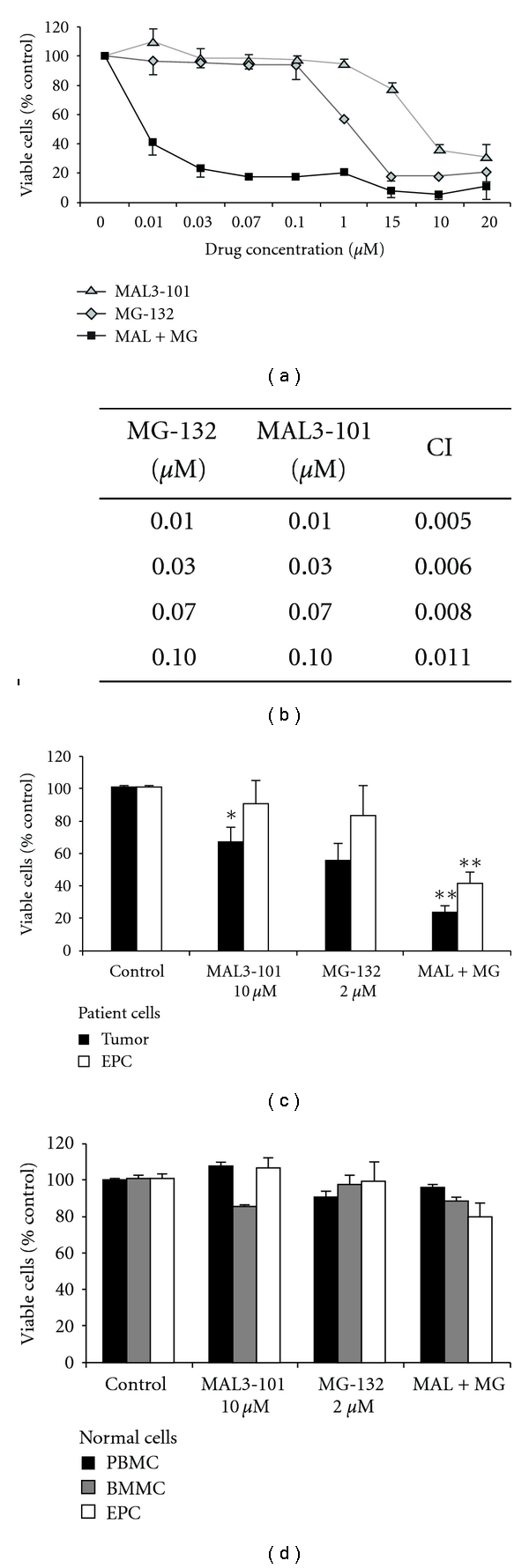
MAL3-101 and MG-132 exhibit synergistic, cytotoxic effects on multiple myeloma tumor cells and endothelial progenitor cells. (a) NCI-H929 cells (1 × 10^5^) were exposed to the indicated concentrations of MAL3-101, MG-132, or a combination of these agents for 40 h, and survival was assessed by an MTS assay; representative data from one of seven independent experiments are shown; error bars represent SDs from replicate data points. Apparent absence of error bars indicates minimal variance. (b) The fraction of nonviable cells compared to control, DMSO-treated cells in this experiment was used for isobologram analysis. In brief, the cellular fraction affected (Fa) was calculated based on the indicated MTS assays using the following formula: Fa = 100 minus the percent of viable cells. Fa values at the indicated drug concentrations were used by CalcuSyn to derive combination index (CI) values, where CI = 1 indicates additive effects, CI < 1 indicates synergy, and CI > 1 indicates antagonism [14]. (c) Bone-marrow-derived tumor cells (black bars) and confluent endothelial progenitor cells (EPCs) (white bars) from multiple myeloma patients were exposed to the indicated concentrations of MAL3-101, MG-132, or their combination, and survival was assessed by an MTS assay. Dunnett's test, after significant one-way repeated-measures analyses of variance (*P* = 0.004 and 0.002 for tumor cells and EPCs, resp.), compared control values to cell viability in cultures treated with MAL3-101, MG-132, or MAL3-101 + MG-132 (*P ≤ 0.05, **P ≤ 0.001). (d) Normal peripheral blood mononuclear cells (PBMCs, black bars), bone marrow mononuclear cells (BMMCs, gray bars), and confluent bone-marrow-derived EPCs (white bars) were exposed to the indicated concentrations of MAL3-101, MG-132, or a combination of these agents, and survival was assessed by an MTS assay.

In MM cells, proteasome inhibition results in the accumulation of misfolded and aggregation-prone proteins, including unassembled IG heavy and light chains, which can induce apoptosis [12, 17, 19, 43]. Therefore, we asked whether MAL3-101 would potentiate the antimyeloma effect of proteasome inhibition. NCI-H929 cells were exposed to a range of MAL3-101 or MG-132 concentrations or to a range of concentrations of the two agents in combination. We found that the IC50 for these agents in combination decreased by up to three orders of magnitude (0.008 *μ*M) when compared to single-agent treatments with either MAL3-101 (IC50 8.3 *μ*M) or MG-132 (IC50 1.7 *μ*M) (Figure 3(a) and Supplementary Table). Notably, when examined alone, each compound was ineffective at the IC50 that was obtained when the compounds were combined. In fact, the synergistic and cytotoxic actions of MAL3-101 and MG-132 on NCI-H929 cells occurred over a range of concentrations (0.01–0.1 *μ*M), as was confirmed by CI values of <1, which indicate strong synergy (Figure 3(b)). As expected, a synergistic increase in apoptosis was also observed in NCI-H929 cells exposed to a combination of the two compounds (Figure S2). 

**Figure 4:**
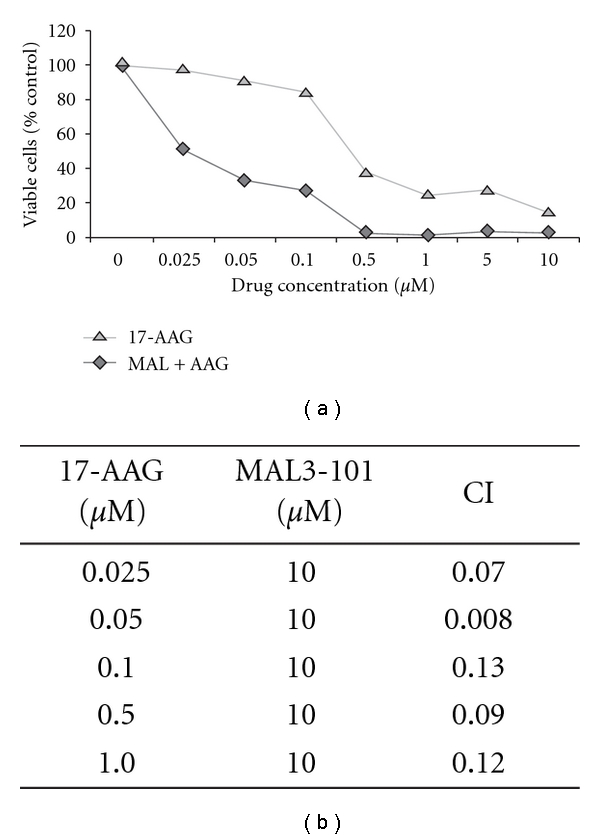
MAL3-101 and 17-AAG exhibit synergistic, cytotoxic effects on the MM cell line NCI-H929. (a) NCI-H929 cells (1 × 10^5^) were exposed to 17-AAG alone or in combination with 10 *μ*M of MAL3-101 for 40 h, and survival was assessed by an MTS assay. Representative data from one of three experiments are shown; error bars represent SDs from replicate data points. An apparent absence of error bars indicates minimal variance. (b) The fraction of nonviable cells compared to control, DMSO-treated cells in this experiment was used for isobologram analysis in CalcuSyn to derive combination index (CI) values, where CI < 1 indicates synergy [14].

 Synergistic antimyeloma effects of MAL3-101 and MG-132 were also confirmed in primary MM cells. As shown in Figure 3(c), the viability of MM cells extracted from the BM was reduced by 33% ± 8  (*P* = 0.02) and 44% ± 10  (*P* = 0.07), respectively, by MAL3-101 and MG-132, and further decreased by 75% ± 4 (*P* = 0.001) when these agents were combined. In addition to its effects on tumor cells, combined treatment with MAL3-101 and MG-132 also exerted synergistic effects on the MM microenvironment. As shown in Figure 3(c) (white bars), the viability of EPCs was reduced by 10% ± 14 (*P* = 0.7) and 16% ± 17 (*P* = 0.3) by MAL3-101 and MG-132, respectively, while their combination decreased EPC viability by 60% ± 7 (*P* = 0.001). The specificity of MAL3-101's effect on MM tumor and microenvironment was indicated by a lack of cytotoxicity in normal control BM, PBMC, and EPC populations (Figure 3(d)). These results are consistent with a previously observed resistance to proteasome inhibition in normal lymphocytes [44] and further indicate the specificity of action of MAL3-101 against tumor cells in MM. Because the Hsp90 molecular chaperone stabilizes oncoproteins, helps maintain MM cell homeostasis, and has synergy with bortezomib *in vitro *[15, 18], we next assessed the effect of MAL3-101 treatment when combined with the antimyeloma Hsp90 inhibitor 17-AAG. When NCI-H929 cells were simultaneously exposed to a single concentration of MAL3-101 somewhat larger than its IC50 (10 *μ*M) and increasing concentrations of 17-AAG, we found that the combination of MAL3-101 and 17-AAG decreased the IC50 for 17-AAG from 0.4 *μ*M to 0.03 *μ*M (Figures 4(a) and 4(b), and Supplementary Table). These results are consistent with the prediction that the antimyeloma effects of Hsp90 inhibition, which causes upregulation of Hsp70 gene expression [15, 18], can be potentiated by simultaneous inhibition of Hsp70 function. 

**Figure 5:**
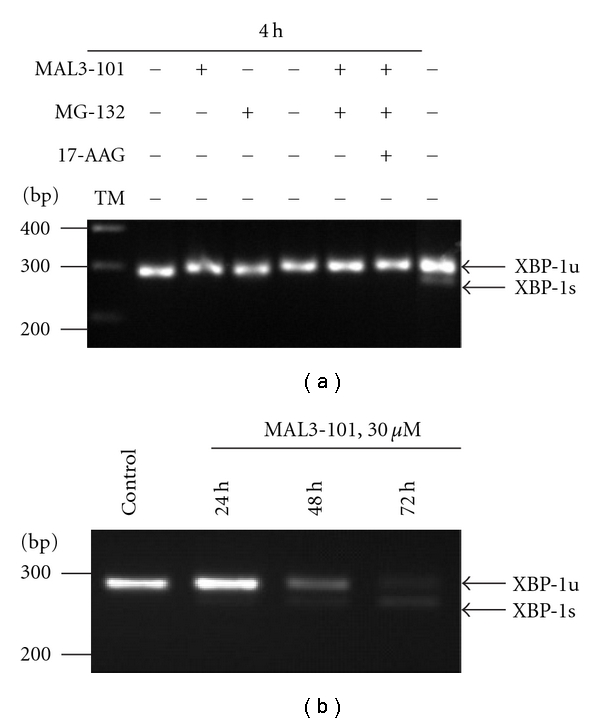
The unfolded protein response is not induced in NCI-H929 cells in response to chaperone and/or proteasome inhibition. RT-PCR analysis was performed to assess XBP-1 mRNA splicing in NCI-H929 cells, and arrows on the right indicate the 285 bp unspliced (XPB-1u) and the 259 bp spliced (XPB-1s) mRNA. (a) NCI-H929 cells were exposed for 4 h to MAL3-101, MG-132, 17AAG, or the indicated combination of the compounds, and total RNA was extracted for RT-PCR amplification. NCI-H929 cells treated for 4 h with 5 *μ*g/mL tunicamycin (TM) as a positive control induced XBP-1 mRNA splicing. (b) NCI-H929 cells were exposed for 0, 24, 48, and 72 h to 30 *μ*M MAL3-101, and total mRNA was extracted for RT-PCR amplification.

 To begin to examine how these compounds individually and in combination impact the viability of NCI-H929 cells, we asked whether the administration of the chemicals would induce the unfolded protein response (UPR). Under conditions of ER stress and UPR induction, the mRNA encoding the X-box binding protein 1 (XBP-1) transcription factor is spliced. This splicing event results in the translation of a 54-kDa XBP-1 spliced (XBP-1s) isoform rather than the 33-kDa XBP-1 unspliced (XBP-1u) isoform. The spliced isoform is essential for plasma cell differentiation and possibly MM progression [6, 12], and XBP-1 splicing represents a sensitive, quantifiable readout for the UPR. At relatively early time points, when UPR induction is maximal, no XBP-1 mRNA splicing was observed after exposure of NCI-H929 cells to MAL3-101, MG-132, or 17AAG at concentrations that had resulted in synergistic cytotoxicity (Figure 5(a)). Consistent with these results, we found that levels of the ER luminal BiP chaperone, whose synthesis is enhanced by UPR activation, did not change in NCI-H929 cells exposed to MAL3-101, MG-132, and/or 17-AAG (data not shown). However, at higher concentrations of MAL3-101 (30 *μ*M, ~4-fold higher than the IC50), a time-dependent increase in XBP-1 mRNA splicing was noted (Figure 5(b)). UPR induction might have resulted from a general Hsp70-dependent block in translation, nascent protein folding, protein transit through the secretory pathway, and/or transcription [12, 42, 45]. 

 Strong experimental evidence indicates that the sensitivity of neoplastic MM cells to proteasome inhibition covaries with monoclonal IG production [17, 19, 46, 47]. Therefore, we measured IG production and trafficking in MAL3-101-treated MM cell lines. When intracellular and secreted IGs were quantified, we observed that the relative amount of IG secretion was highest in NCI-H929 cells, which also demonstrated the highest sensitivity to MAL3-101-induced growth inhibition (Table 1). These data suggest that the sensitivity of MM cells to Hsp70 inhibition arises from the added cellular stress of producing and secreting monoclonal IGs. 

 To determine the feasibility of quantifying the MAL3-101-mediated inhibition of MM cell growth in response to MAL3-101 *in vivo*, we used the subcutaneous NCI-H929 tumor (plasmacytoma) xenograft in NSG recipient mice [38]. Treatments with vehicle (DMSO and PBS), or with 40 mg/kg MAL3-101 and 1 mg/kg PS-341 (bortezomib), or with a combination of 40 mg/kg MAL3-101 and 1 mg/kg PS-341 i.p., were started 24 h after s.c. tumor cell injections. Dose and schedule of drugs were supported by preliminary observations of tumor growth in response to treatment with MAL3-101 at an alternate dose and schedule (Figure S3). 

**Figure 6:**
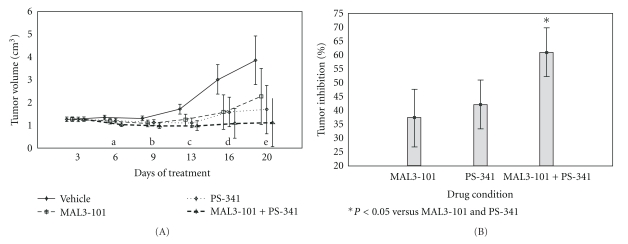
Effect of MAL3-101 and PS-341 on myeloma cell growth *in vivo. *(A) shows repeated measures as condition effect in NSG mice which were inoculated subcutaneously in the right flank with 3 × 10^7^ NCI-H929 cells. Mice received 40 mg/kg MAL3-101, 1 mg/kg PS-341, a combination of these agents, or the vehicle twice weekly i.p. (*n* = 16). Tumor volume assessments were performed on indicated days. Overall repeated measures by treatment condition were observed (*F*(15,50) = 4.89; *P* = 0.00001), and MAL3-101 and PS-341 each delayed tumor growth over 20 days from initial treatment; however, in combination, MAL3*­*101 and PS-341 had a greater effect than the single treatments by delaying tumor progression and reducing tumor size. Vertical bars denote 0.95 confidence interval. a–e refer to results of ANOVAs for effect of treatment by day on tumor inhibition. (a) Overall condition effect on day 6: *F*(3,10) = 5.40; *P* = 0.018; Tukey HSD: vehicle > MAL3-101 and PS-341 combination. (b) Overall condition effect on day 9: *F*(3,10) = 5.25; *P* = 0.02; Tukey HSD: vehicle > MAL3-101 and PS-341 combination. (c) Overall condition effect on day 13: *F*(3,10) = 10.57; *P* = 0.002; Tukey HSD: vehicle > MAL3-101 and PS-341 combination; vehicle > PS-341. (d) Overall condition effect on day 16: *F*(3,10) = 8.16; *P* = 0.004; Tukey HSD: vehicle > MAL3*­*-101 and PS-341 combination; vehicle > PS-341. (e) Overall condition effect on day 20: *F*(3,10) = 7.33; *P* = 0.01; Tukey HSD: vehicle > MAL3*­*-101 and PS-341 combination; vehicle > PS-341. (B) The bar graph shows the computed mean percentage difference for each treatment versus vehicle. An overall condition effect was noted (*F*(2,7) = 10.25; *P* = 0.008). Treatment with combination of PS-341 and MAL3-101 was significantly more effective on percent tumor inhibition in comparison to PS-341 or MAL3-101 alone (Tukey's HSD test, *P* = 0.02; *P* = 0.008, resp.), although each individual treatment was not significantly different from each other. *indicates statistical significance. Vertical bars denote 0.95 confidence interval.

 An overall repeated measures by treatment condition was observed (*F*(15,50) = 4.89; *P* = 0.00001) as shown in Figure 6(A). Compared to vehicle-treated controls, MAL3-101 and PS-341 each delayed tumor growth over 20 days from the initial treatment; however, in combination, MAL3*­*-101 and PS-341 had a greater effect than the single treatments by delaying tumor progression and reducing tumor size. Significant ANOVA effects were noted for Days 6, 9, 13, 16, and 20 (Figure 6(A)). For days 6–20, tumor volume was significantly greater for vehicle than for combination treatment with MAL3-101 and PS-341, and for days 13, 16, and 20, tumor volume was significantly greater for vehicle than PS-341 alone. No differences were noted for vehicle versus MAL3-101 alone. In order to compare the condition effects on tumor progression, we computed the mean percentage difference for each treatment versus vehicle as shown in Figure 6(B). An overall condition effect was noted (*F*(2,7) = 10.25; *P* = 0.008). Analyses also showed that combined treatment with PS-341 and MAL3-101 was significantly more effective on percent tumor inhibition in comparison to treatment with PS-341 or MAL3-101 individually (Tukey's HSD test, *P* = 0.02; *P* = 0.008, resp.) (Figure 6(B)). In summary, the *in vivo *data are in accordance with our *in vitro *results and indicate that simultaneous inhibition of the proteasome and Hsp70 by MAL3-101 has a stable suppressive effect on tumor growth detected as early as day 6 in our MM model.

## 4. Discussion

The focus of this study was to measure the impact of MAL3-101 on *in vitro* and *in vivo *growth of MM cells and to predict the clinical effectiveness of this compound against MM. Our results provide evidence that MAL3-101 is cytotoxic to MM cells, and that synergistic effects are observed when this compound is used in combination with Hsp90 or proteasome inhibitors. 

 In MM, the protein-folding machinery is overloaded due to increased IG synthesis and secretion, and a significant population of the IGs may misfold; in fact, some MM cells produce unassembled IG single chains. The concentration of these proteins in the ER may be decreased by being retrotranslocated back to the cytoplasm, where they are degraded by the proteasome, a process referred to as ER-associated degradation (ERAD) [48, 49]. Since IGs fold in the ER and traffic through the secretory pathway, proteasome inhibition results in an increased load of misfolded proteins in the ER, thus triggering apoptosis. Because Hsp70 homologs in the ER and in the cytoplasm play critical roles in ERAD [28, 48, 49], it may not be surprising that we observed synergistic cytotoxic effects on MM cell survival when the proteasome and Hsp70 were simultaneously inhibited. We also observed synergistic cytotoxicity in MM cells by a combined inhibition of Hsp70 and Hsp90, further supporting the notion that MM cells are susceptible to treatments that compromise protein quality control. 

 The most MAL3-101-responsive cell line, NCI-H929, is a high secretor of monoclonal IG [50] and is consistent with other published data that IG load correlates with sensitivity to inhibitors of the quality control machinery [17, 43]. Even though the lack of UPR induction in MM cells has been previously reported [12], we envisioned that this response might be triggered when both Hsp70 and the proteasome were inhibited since ERAD substrates should accumulate. The lack of UPR induction by MAL3-101, Hsp90, and proteasome inhibitors at the low concentrations that induced synergistic apoptosis may be multifactorial: While NCI-H929 cells synthesize and secrete large amounts of IGs, they may be unable to induce a sustained UPR. Alternatively, apoptosis may occur by pathways independent of UPR activation, including autophagy [45] or aggresome disposal [14]; therefore, it will be important to better define at the molecular level the mechanisms that regulate cell stress responses [51]. Further work is necessary to establish whether MAL3-101 has effects on pathways that are upregulated in MM cells [14, 45]. Nevertheless, Davenport and colleagues [15] showed that proteasome inhibition in MM cell lines by bortezomib does not necessarily result in the production of XBP1s, but ER stress and activation of the intrinsic apoptotic pathway are evident. Moreover, 2 h after exposure to 17-AAG, there was a rapid initiation of the UPR, which leads to ER stress and activation of intrinsic apoptotic pathways. More recently, the same group discovered that the 17AAG-induced UPR was synergistically increased by Hsp72 inhibition in MM cell lines [21]. By analogy, we found that the level of XBP1s increased after exposure to MAL3101 after 48 h. However, at effective antimyeloma concentrations, we did not observe XBP1s when MAL3-101 and 17-AAG were coadministered, unless much higher concentrations and a prolonged incubation were used. We suggest that the different effects observed may result from the nature of the compounds that were employed to inhibit Hsp70, and/or that the activities of unique Hsp70 family members may be differentially affected by these chemicals. 

 A critical result of our studies is the demonstration of the synergistic effects of Hsp70 and proteasome inhibition on the MM microenvironment. We and others have observed IG gene rearrangement in MM EPCs [40, 52]; therefore, protein misfolding and the ensuing ER stress response may determine the susceptibility of tumor endothelial cells, in at least a population of patients, to Hsp70 and proteasome inhibition. This possibility is supported by a lack of sensitivity to MAL3-101 in normal bone marrow, lymphocytes, and endothelial cells, most of which do not produce IG. As a result, the clinical application of MAL3-101 in MM may not only help overcome drug resistance by potentiating the effects of other protein quality control inhibitors, but it may also limit MM growth via inhibition of tumor angiogenesis. 

 The antimyeloma effects of MAL3-101 were apparent at concentrations similar to those used with 15-deoxyspergualin, a Hsp70 modulator with a KD for Hsp70 of ~5 *μ*M. 15-Deoxyspergualin has proven successful in clinical trials [53]; however, this compound is a nonspecific chaperone inhibitor and may exhibit other off-pathway effects [54]. Our results are also in agreement with recent data showing strong expression of Hsp70 expression in MM cell lines at baseline and significant upregulation after extracellular matrix (ECM) adhesion [26]. In the same report, it was also demonstrated that inhibition of Hsp70 gene expression induced a reduction in tumor cell adhesion to ECM followed by an increase in apoptosis in MM cells. 

 In summary, we suggest that MAL3-101, an Hsp70 modulator that specifically binds to Hsp70 and affects its interaction with cochaperones [1, 28, 55], may provide a viable means to enhance the antimyeloma effects of both proteasome and Hsp90 inhibitors. Coadministration strategies may reduce drug resistance in MM treatments and allow those patients who currently cannot tolerate bortezomib [13] to benefit from the drug because lower concentrations can be used in conjunction with an MAL3-101-like molecule. In addition to direct antitumor properties, MAL3-101 may affect the microenvironment by targeting the microvasculature. Our experiments using a xenograft plasmacytoma model show that tumor cell growth *in vivo *is also delayed and reduced by MAL3-101, and that this inhibitory effect is synergistic for proteasome inhibition which was observed within the first week after combination treatment was initiated. In this model, because we started treatment with compounds within 24 h of tumor initiation, the findings may possibly be translated clinically to a restricted effect at earlier stages of the disease prior to extensive skeletal involvement. It is also possible that combinations containing higher doses of MAL3-101 with proteasome inhibition may be required to control larger tumors and MM at its later stages. The need to elucidate the pharmacokinetic properties of MAL3-101 is underscored by these results for optimization of dose and frequency schedule of MAL3-101 exposure. Particularly important will be the measurement of plasma levels as well as rate and extent of clearance of the compound when determining effects versus toxicity. In the latter regard, we do know that concentrations up to 160 mg/kg i.p. have been tolerated without toxicity (data not shown). The results presented here strongly suggest that more detailed studies of MAL3-101 pharmacokinetics are warranted.

## Supplementary Material

Comparison of the inhibitory concentrations (IC_50_) of indicated combinations of MAL3-101, MG-132 and 17-AAG in NCI-H929 cells (compared to DMSO-treated control cells) are shown in Table S1. Structures of MAL3-101 and MAL3-51 are shown in Figure S1. Dot plots shown in Figure S2 illustrate apoptosis in NCI-H929 cells caused by exposure to indicated concentrations of MAL3-101, MG-132, or their combination obtained by dual Annexin V and propidium iodide (PI) staining and flow cytometry.
Figure S3 shows preliminary *in vivo* tumor progression experiments where NSG mice were treated i.p. 20 mg/kg MAL3-101 either 24 h after tumor inoculation (*n*=1) or 8 d after tumor inoculation (*n*=1) as indicated. Each drug schedule was controlled with an animal treated with vehicle. All mice (*n*=4) were inoculated subcutaneously in the right flank with 3 × 10^7^ NCI-H929 cells. Treatment with 20 mg/kg MAL3-101 or vehicle was given twice weekly via i.p. The day of tumor inoculation is considered Day 0 on the **x**-axis. Tumor volume analyses for days 0, 14 and 29 after tumor inoculation are shown. Mean tumor volumes are shown for vehicle-treated animals.

## Figures and Tables

**Table 1:** MM cell line NCI-H929 is a high secretor of monoclonal IG. Multiple myeloma (MM) cell lines were grown at 1 × 10^6^ cells/mL for 16 h, and 500 ng total protein from cell lysates and 500 ng from serum-free supernatants were used in ELISAs to determine intracellular and secreted immunoglobulin (IG) light-chain (LC) levels, respectively. Relative LC secretion is shown as the proportion of secreted versus intracellular LC levels. Data are presented as the mean ± SD from 3 independent experiments.

MM cell line	Light chain (LC) produced	Secreted LC (ng/10^6^ cells)	Intracellular LC (ng/10^6^ cells)	Relative LC secretion (secreted/intracellular)
NCI-H929	*κ*	505 ± 22	93 ± 9.9	5.46 ± 0.4
U266	*λ*	639 ± 30	387 ± 32	1.66 ± 0.2
RPMI-8226	*λ*	56 ± 51	1271 ± 18	0.16 ± 0.1
