# Supplementary figures and images for: Antimyeloma Effects of the Heat Shock Protein 70 Molecular Chaperone Inhibitor MAL3-101
Source: J Oncol. 2011 Sep 29;2011:232037. doi: 10.1155/2011/232037 (PMC3184436; doi:10.1155/2011/232037)

## Supplementary Fig. S1

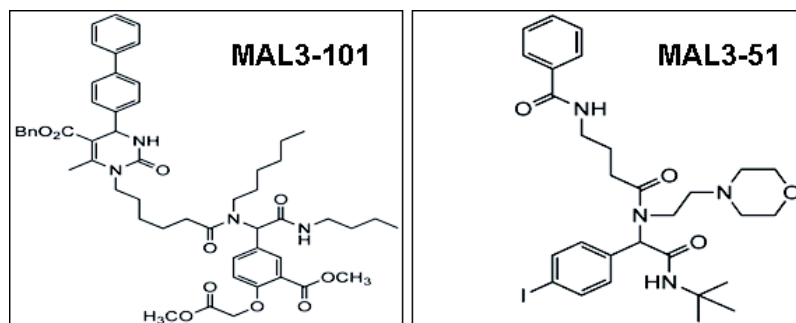

Supplement: Supplementary file 1 — Comparison of the inhibitory concentrations (IC50) of indicated combinations of MAL3-101, MG-132 and 17-AAG in NCI-H929 cells (compared to DMSO-treated control cells) are shown in Table S1. Structures of MAL3-101 and MAL3-51 are shown in Figure S1. Dot plots shown in Figure S2 illustrate apoptosis in NCI-H929 cells caused by exposure to indicated concentrations of MAL3-101, MG-132, or their combination obtained by dual Annexin V and propidium iodide (PI) staining and flow cytometry. Figure S3 shows preliminary in vivo tumor progression experiments where NSG mice were treated i.p. 20 mg/kg MAL3-101 either 24 h after tumor inoculation (n=1) or 8 d after tumor inoculation (n=1) as indicated. Each drug schedule was controlled with an animal treated with vehicle. All mice (n=4) were inoculated subcutaneously in the right flank with 3 × 10^7^ NCI-H929 cells. Treatment with 20 mg/kg MAL3-101 or vehicle was given twice weekly via i.p. The day of tumor inoculation is considered Day 0 on the x-axis. Tumor volume analyses for days 0, 14 and 29 after tumor inoculation are shown. Mean tumor volumes are shown for vehicle-treated animals. [file 232037.f1.pdf]

## Supplementary Fig. S2

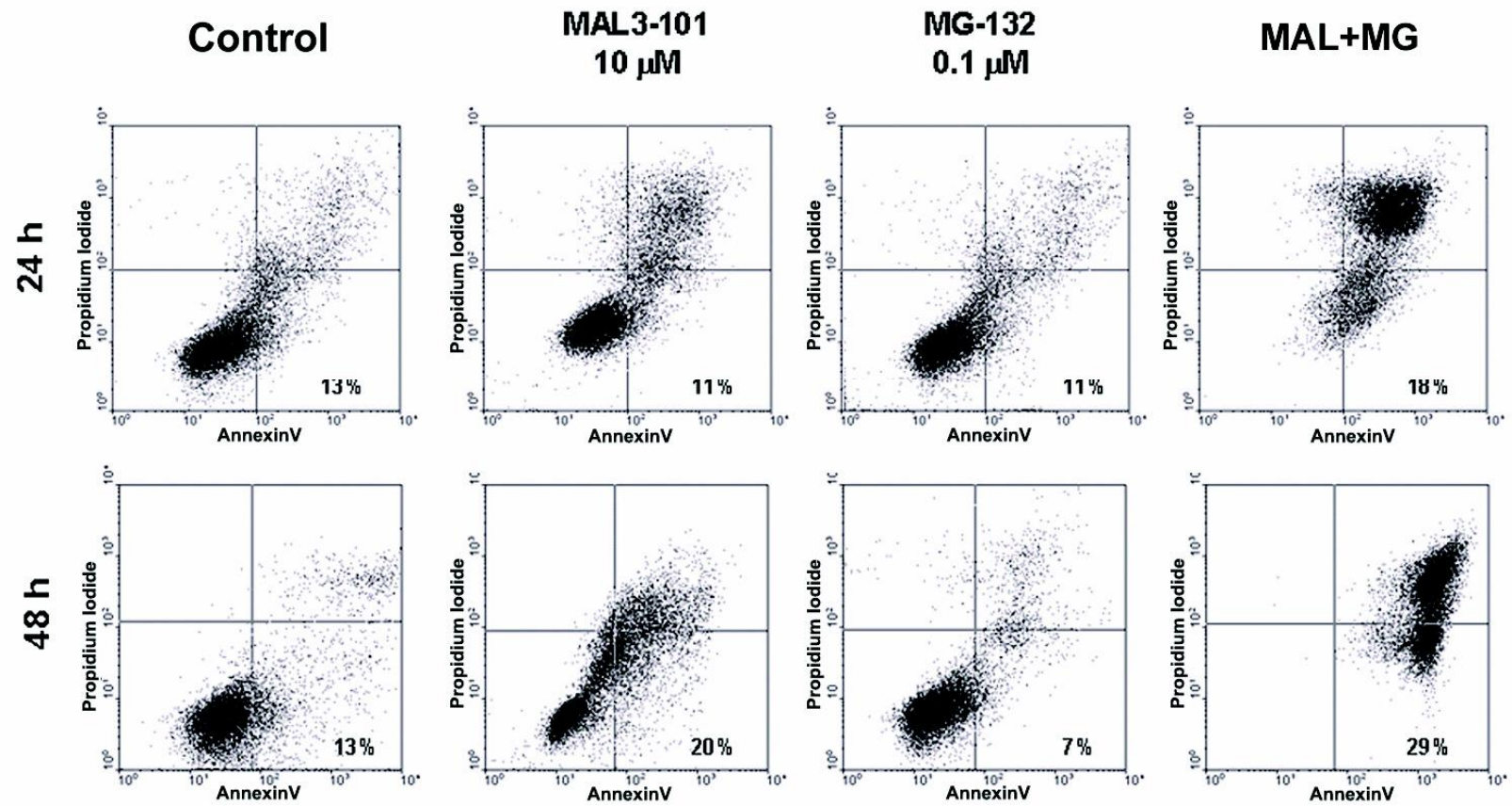

Supplement: Supplementary file 2 [file 232037.f2.pdf]

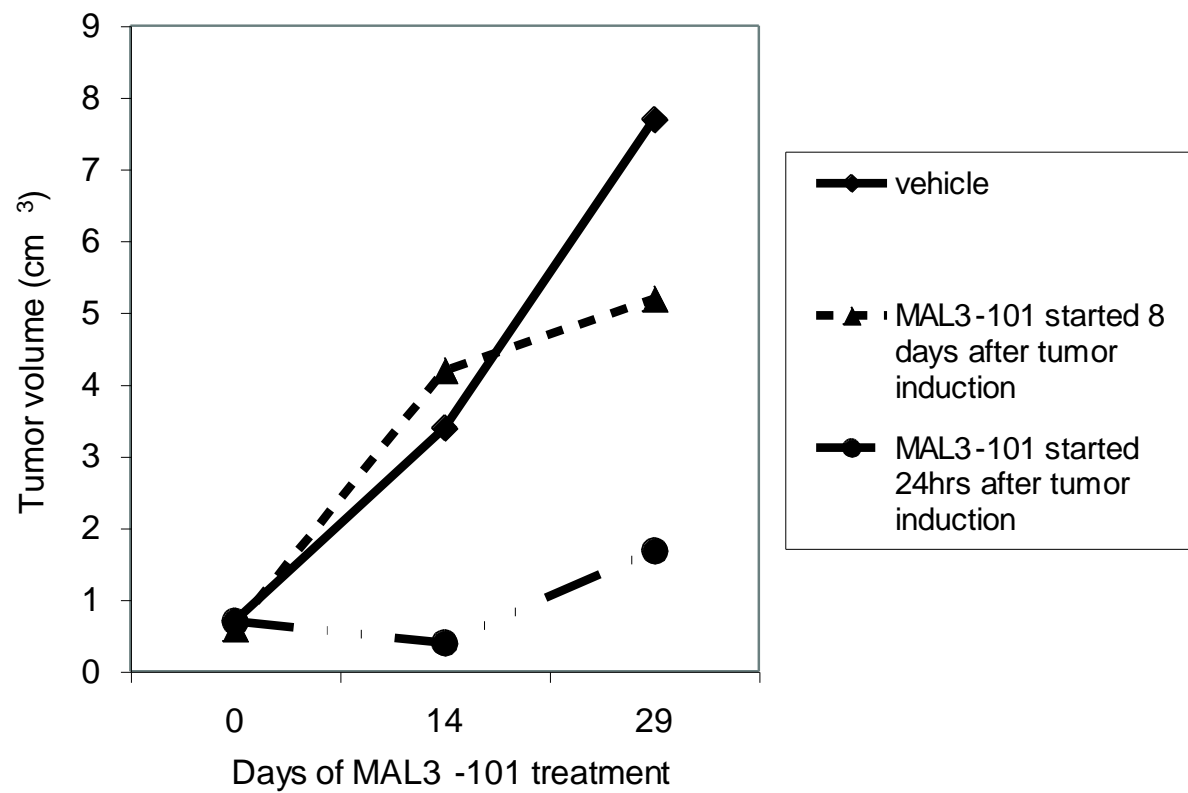

Supplement: Supplementary file 3 [file 232037.f3.pdf]
