# Supplementary material for: Antimyeloma Effects of the Heat Shock Protein 70 Molecular Chaperone Inhibitor MAL3-101
Source: J Oncol. 2011 Sep 29;2011:232037. doi: 10.1155/2011/232037 (PMC3184436; doi:10.1155/2011/232037)
Supplement: Supplementary file 4 [file 232037.f4.pdf]

Supplementary Table

Treatment of NCI-H929	IC_{50} (! M) at 40 h
<u>Single agents</u>	
MAL3-101	8.3
MG-132	1.7
17-AAG	0.40
<u>Combinations</u>	
MAL3-101 + MG-132	0.008
MAL3-101 + 17-AAG	0.03
